# Supplementary material for: Endoscopic Contrast-Enhanced Ultrasound and Fine-Needle Aspiration or Biopsy for the Diagnosis of Pancreatic Solid Lesions: A Systematic Review and Meta-Analysis
Source: Cancers (Basel). 2024 Apr 25;16(9):1658. doi: 10.3390/cancers16091658 (PMC11083840; doi:10.3390/cancers16091658)
Supplement: Supplementary file 1 [file cancers-16-01658-s001.zip › cancers-2960475-supplementary.pdf]

Table S1: Search Terms and Search Strategy

Pubmed	
**Concept 1: Pancreatic neoplasm**	("Pancreatic Cyst"[Mesh]) OR "Pancreatic Neoplasms"[Mesh] OR "Pancreatic Adenocarcinoma") OR "Pancreatitis"[Mesh] OR "Pancreatic NET" OR "Pancreatic Neuroendocrine Tumor"
**Concept 2: Ultrasound**	ultrasonograph* OR ultrasound OR sonogra* OR endosonograph*
**Concept 3: Contrast Enhanced Ultrasound**	("contrast-enhanced" OR "contrast-enhanced" OR "contrast medium" OR "contrast agent*" OR "echo-enhanced") OR "CEUS" OR "ECEUS"
**Combination of concepts**	(Concept 1) AND (Concept 2) AND (Concept 3)

Embase	
**Concept 1: Pancreatic neoplasm**	"pancreatic cyst":ab,ti OR "pancreatic neoplasm":ab,ti OR "pancreatic adenocarcinoma":ab,ti OR "pancreatic net" OR "pancreatic neuroendocrine tumor":ab,ti
**Concept 2: Ultrasound**	'ultrasonograph*':ab,ti OR 'ultrasound':ab,ti OR 'sonograph*':ab,ti OR 'endosonograph*':ab,ti
**Concept 3: Contrast Enhanced Ultrasound**	'echo-enhanced':ab,ti OR 'contrast agent':ab,ti OR 'contrast enhanced':ab,ti OR 'ceus':ab,ti OR 'eceus':ab,ti
**Combination of concepts**	(Concept 1) AND (Concept 2) AND (Concept 3)

Web of Science	
**Concept 1: Pancreatic neoplasm**	TS = "Pancreatic Cyst" OR "Pancreatic adenocarcinoma" OR "Pancreatic Neoplasm" OR "Pancreatic NET" OR "Pancreatic neuroendocrine tumor"
**Concept 2: Ultrasound**	TS = "Ultrasonograph*" OR "ultrasound" OR "sonogra*" OR "endosonograph*"
**Concept 3: Contrast Enhanced Ultrasound**	TS = "contrast enhanced" OR "contrast agent" OR "echo-enhanced" OR "CEUS" OR "ECEUS"
**Combination of concepts**	(Concept 1) AND (Concept 2) AND (Concept 3)
